# Upregulation of microRNA-126-5p is associated with drug resistance to cytarabine and poor prognosis in AML patients

**DOI:** 10.3892/or.2015.3839

**Published:** 2015-03-06

**Authors:** YOSHIHIKO SHIBAYAMA, TAKESHI KONDO, HIROKI OHYA, SHIN-ICHI FUJISAWA, TAKANORI TESHIMA, KEN ISEKI

**Affiliations:** 1 Education Research Center for Clinical Pharmacy, Graduate School of Pharmaceutical Sciences, Hokkaido University, Sapporo, Hokkaido 060-0812, Japan; 2 Department of Hematology, Graduate School of Medicine, Hokkaido University, Sapporo, Hokkaido 060-0812, Japan; 3 Laboratory of Clinical Pharmaceutics and Therapeutics, Graduate School of Pharmaceutical Sciences, Hokkaido University, Sapporo, Hokkaido 060-0812, Japan; 4 Department of Clinical Laboratory, Hokkaido University Hospital, Hokkaido University, Sapporo, Hokkaido 060-0812, Japan

**Keywords:** acute promyelocytic leukemia, Akt, cytarabine, Klotho, miR-126

## Abstract

MicroRNAs (miRs) have been shown to negatively regulate gene expression by binding to mRNAs, and they play an important role in various physiological processes and ma lignancies. A previous study identified mature miR-126-3p as an onco-microRNA that is generated from the pre-microRNA, miR-126. Although miR-126 also generates mature miR-126-5p, its function is less clear. In the present study, the relationship between miR-126-5p/3p expression levels and overall survival in 109 patients with acute myeloid leukemia (AML) who received intensive therapy were evaluated. Higher expression levels above the median value of miR-126-5p/3p were correlated with a poorer overall survival. The hazard ratio and 95% confidence intervals (95% CI) for the higher expression group relative to the lower expression group of miR-126-5p/3p were 2.098 (95% CI: 1.075–4.228) and 1.958 (95% CI: 1.001–3.927), respectively. An interaction was not observed between the hazard ratios of miR-126-5p and miR-126-3p (p=0.73). Transfection of the mimic miR-126-5p into the AML cell line, KG-1, resulted in a decrease in the sensitivity to cytarabin and the expression level of *Klotho* mRNA as well as the elevation in the phosphorylation of Akt. The results of the present study demonstrated that higher expression levels of miR-126-5p/3p in patients with AML resulted in a poorer prognosis. Furthermore, miR-126-5p elevated the phosphorylation of Akt.

## Introduction

Acute myeloid leukemia (AML) is characterized by the clonal proliferation of myeloid precursors. Leukemic blasts or immature forms accumulate in the bone marrow, peripheral blood and occasionally in other tissues, resulting in variable reductions in the production of normal blood cells. Although some clinical factors such as age, performance status or a history of prior chemotherapy have been associated with the outcomes of patients with AML, the most important factor in predicting the risk of relapse has been chromosomal abnormalities detected at diagnosis (1–4). Proto-oncogene mutations are an important consideration in the risk stratification of patients with AML (5). The mutational diagnosis of *NMP1*, *PML-RARA*, *CBFB-MYH11*, *FLT3* and *RAS* has been applied to clinical practice and has consequently impacted diagnoses, risk assessments and also guidance related to available therapies. Changes in the expression of specific genes (e.g., *ABCB1*, *BAALC*, *ERG*, *MN1*, *JAK2* and *WT1*) appear to influence the prognoses of molecular subsets of patients with AML (3).

MicroRNAs (miRs) have been shown to negatively regulate gene expression by binding to the mRNAs of protein-coding genes, thereby degrading or blocking their translation. MicroRNAs are also known to play an important role in various physiological and pathological processes, such as apoptosis, cell proliferation and differentiation, which indicates their functionality in carcinogenesis as tumor-suppressor genes or proto-oncogenes (6). MicroRNA expression profiling has been used to distinguish between myeloid and lymphoid leukemia, as well as distinct cytogenetic subtypes of AML based on the upregulation or downregulation of specific miRNAs. MicroRNA expression signatures have also been correlated with recurrent molecular aberrations in AML (5). miR-126-3p is known to play an important role in various physiological processes. A previous study reported that miR-126-3p (accession no. MIMAT0000445) inhibited cell apoptosis and increased cell viability in AML cells (7). Mature miR-126-3p is generated from the stem-loop sequence miR-126 (accession no. MI0000471). Although miR-126 also generates mature miR-126-5p (accession no. MIMAT0000444), its function is less clear.

Bioinformatics, microRNA.org, predicted that miR-126-5p targets *Klotho*. The klotho gene functions as an aging suppressor gene that extends the life-span. Recent studies have revealed that Klotho plays an important role in cancer tumorigenesis (8). The Klotho protein can regulate multiple growth factor signaling pathways, including IGF-1 and Wnt, as well as the activity of multiple ion channels (9,10). The overexpression of Klotho was previously shown to inhibit cancer cell proliferation and may act as a potential therapeutic strategy in cancers (8).

It currently remains unclear whether miR-126-5p is involved in the survival of patients with AML. The inhibitory effects of miR-126-5p on the expression of *Klotho* mRNA have not yet been elucidated in detail. We herein investigated the survival benefits of miR-126-5p in patients with AML and the inhibitory effects of a possible target mRNA of miR-126-5p and *Klotho*.

## Materials and methods

### Reagents

The TaqMan gene expression assays, siPORT *Neo*FX Transfection Agent and TRIzol reagent were obtained from Life Technologies (Carlsbad, CA, USA). The QuantiTect primer assay, miScript primer assay, miScript reverse transcription kit, synthetic microRNA mimic, QIAamp RNA Blood Mini kit, AllStars negative control siRNA and miScript SYBR-Green PCR kit were purchased from Qiagen (Valencia, CA, USA). Real-time PCR Master Mix Thunderbird SYBR qPCR Mix and reverse transcriptase, ReverTra Ace were purchased from Toyobo Co., Ltd., (Osaka, Japan). Protease inhibitor cocktail tablets (Complete, EDTA-free) were purchased from Roche Diagnostics GmbH (Buckinghamshire, UK). RPMI-1640 medium was purchased from Sigma-Aldrich (St. Louis, MO, USA). The human acute myelogenous leukemia cell line, KG-1 (JCRB9051), and the human myelogenous leukemia cell line, K562 (JCRB0019), were purchased from the Japanese Collection of Research Bioresources Cell Bank (Osaka, Japan). This cell line was tested and authenticated by the JCRB Cell Bank. The human proximal tubular cell line, HK-2, was purchased from the American Type Culture Collection. Mouse monoclonal antibodies specific for anti-β-actin (AC-74), Akt1 (5c10), anti-phosphorylated Akt1 (104A282), anti-caspase-3 (NB500-210SS) and anti-JAK2 (1C1) were purchased from Sigma-Aldrich, Enzo Life Sciences (Farmingdale, NY, USA), Abcam (Cambridge, UK) and GeneTex (San Antonio, TX, USA), respectively. Rabbit polyclonal antibodies specific for phosphorylated-JAK2, STAT3, phosphorylated-STAT3, STAT5, phosphorylated-STAT5 and Klotho were purchased from GeneTex. The synthetic *Klotho* siRNA (sense, GGAUUGACCUUGAAUUUAATT and antisense: UUAAAUUCAAGGUCAAUCCTT), PCR primers for *Klotho* (sense, GCTCTCAAAGCCCACATACTG and antisense, GCAGCATAACGATAGAGGCC) and PCR primers for *ACTB* actin, β (β-actin sense, TGACGTGGACATCCG CAAAG and antisense, CTGGAAGGTGGACAGCGAGG) (11,12) were obtained from Fasmac Co., Ltd. (Atsugi, Japan). All other reagents were purchased from Wako Pure Chemical Industries (Osaka, Japan).

### Sampling of bone marrow

Bone marrow from 109 patients diagnosed with AML was obtained from the North Japan Hematology Study Group. The characteristics of the patients who received intensive therapy are described in Table I. Chromosomal aberrations were identified at participating centers using standard procedures for bone marrow morphology, cytochemistry and flow cytometry, along with cytogenetics using FISH and/or RT-PCR. An informed written consent was obtained from all the patients that participated in this study. The present study was approved by the Ethics Committee of the Graduate School of Medicine, Hokkaido University.

### Cell culture and transfection assays

The human myeloid leukemia cell lines, KG-1 and K562, were grown in RPMI-1640 medium. The human proximal tubular cell line, HK-2, was grown in DMEM/Ham's F-12 medium. The medium was supplemented with 10% fetal bovine serum, 2 mM glutamine, and 100 units/ml of penicillin at 37°C in a 5% CO_2_ humidified atmosphere. The synthetic miRNA inhibitor and precursor were transfected using siPORT *Neo*FX transfection agent according to the manufacturer's protocol. In 96-well plates, 3 pmol of the inhibitor or precursor was transfected using 0.3 *μ*l of the siPORT *Neo*FX and the cells were harvested 72 h later for the 3-(4,5-dimethylthiazol-2-yl)-2,5-diphenyl tetrazolium bromide (MTT) assay. Cytarabine and idarubicin were added to the wells and the cells were harvested at 120 and 48 h, respectively. The cells were incubated with MTT solution, and incubation was stopped with 20% sodium dodecyl sulfate solution. The 96-well plates were shacken overnight in the dark and the absorbance was then determined at 570 nm.

### Real-time quantitative reverse transcription-polymerase chain reaction (RT-PCR) analysis

The total RNA was isolated from the bone marrow using the QIAamp RNA Blood Mini kit according to the manufacturer's instructions. Single-stranded cDNA was synthesized by reverse-transcriptase using ReverTra Ace and single-stranded cDNA for the miRNA analysis was also synthesized by reverse-transcriptase using the miScript reverse transcription kit according to the manufacturer's instructions. Real-time PCR was performed on the LightCycler 480 ΙΙ System (version 1.5; Roche Diagnostics GmbH, Mannheim, Germany) using TaqMan gene expression assays and Thunderbird qPCR Mix or the miScript SYBR-Green PCR kit according to the manufacturer's instructions. The comparative quantification cycle threshold (Cq) method was used to determine the relative expression levels of the target genes. Cq values were calculated by the 2nd derivative maximum method. Glucose-phosphate isomerase (*GPI*) and RNU6B (U6) were analyzed as reference genes for mRNA and miR, respectively (13,14). The cycle number difference (ΔCq = reference genes - target genes) was calculated for each replicate. Relative target gene expression values were calculated using the mean of ΔCq from 3 replicates, *μ*(ΔCq) =Σ(ΔCq)/3 and expressed as 2*^μ^*^(ΔCq)^ (13).

### Electrophoresis and western blot analysis

Protein sampling and the western blot analysis were performed as described previously (15). Whole cell homogenates (β-actin, 5 *μ*g; other proteins, 20 *μ*g) were separated using 10% SDS polyacrylamide gels and proteins were then transferred to a nitrocellulose membrane. The membranes were incubated with either the AC-74 (10,000-fold dilution), 5C10 (1,000-fold dilution), 104A282 (1,000-fold dilution), 1C1 (500-fold dilution) or NB500-210SS (2,000-fold dilution) monoclonal antibody and with the rabbit polyclonal antibody (1,000-fold dilution) in the blocking solution. The detection method has been described in detail previously (15).

### Statistical analysis

Comparisons between 2 groups were performed with the Mann-Whitney U test or the Student's t-test. Comparisons between 3 groups were performed with the Tukey-Kramer test. Categorical variables were analyzed with the two-tailed χ^2^ test or Fisher's exact test (expected frequency <5). Survival was plotted with the Kaplan-Meier curves, using the interval from the date of the AML diagnosis to death or to the last contact. Comparisons between each group were performed with the log-rank test. Overall survival was evaluated using the Cox proportional hazards model. All indicated P-values were two-sided: P<0.05, P<0.01 and P<0.001.

## Results

### Relationships between the overall survival and the expression levels of miR-126-5p and miR-126-3p

**Figure 1:**
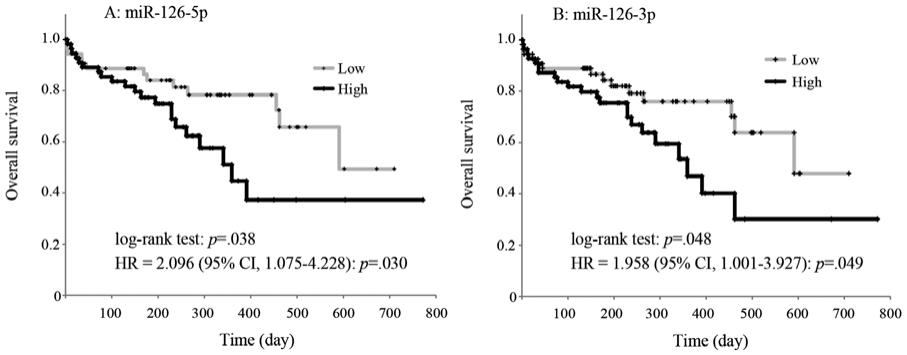
Kaplan-Meier overall survival curves for patients with AML according to the low and high expression of miR-126-5p and miR-126-3p. Kaplan-Meier plots showing estimates of overall survival probabilities grouped based on the miR-126-5p expression levels in a completely independent set of 109 AML patients. (A) The black line curve represents the samples with high (above median, 2^−ΔΔCq^ value >1.1×10^−4^, n=55) miR-126-5p expression levels, whereas the dotted line curve corresponds to the samples with low (below median, 2^−ΔΔCq^ value <1.1×10^−4^, n=54) miR-126-5p expression levels. (B) The black line curve represents the samples with high (above median, 2^−ΔΔCq^ value >2.0×10^−4^, n=54) miR-126-3p expression levels, whereas the dotted line curve corresponds to the samples with low (below median, 2^−ΔΔCq^ value <2.0×10^−4^, n=54) miR-126-3p expression levels. Comparisons between each group were performed with the log-rank test. Cox proportional hazards models also estimated a significantly higher HR of miR-126-5p and miR-126-3p in the high group: miR-126-5p: HR=2.096 (95% CI, 1.075–4.228), p=0.030 and miR-126-3p: HR=1.958 (95% CI, 1.001–3.927), p=0.049. In the Cox proportional hazard regression model, the ‘miR-126-5p x miR-126-3p’ interaction was not significant (p=0.73). CI, confidence interval; AML, acute myeloid leukemia; HR, hazard ratio.

The relationship between miR-126-5p expression level and the overall survival duration in 109 patients who received intensive therapy was evaluated. Patients were divided into two groups: the high group expressed greater amounts of miR-126-5p than the median value of miR-126-5p expression while the low group expressed less. No significant differences were observed in the patient characteristics or gene mutation statuses of the high and low groups (Tables I and II). No significant differences were observed in the expression levels of RNA-related abnormalities in AML between the two groups (Table III). Higher miR-126-5p expression levels (more than the median value) correlated with a poorer probability of overall survival (Fig. 1A). Higher miR-126-3p expression levels also correlated with a poorer overall survival (Fig. 1B). Cox proportional hazards models also estimated a significant higher hazard ratio (HR) of miR-126-5p and miR-126-3p in the high expression group (Fig. 1). In the Cox proportional hazard regression model, the ‘miR-126-5p × miR-126-3p’ interaction was not significant (p=0.73).

### Effects of miR-126-5p transfection on drug sensitivity, Klotho expression and Akt phosphorylation

**Figure 2:**
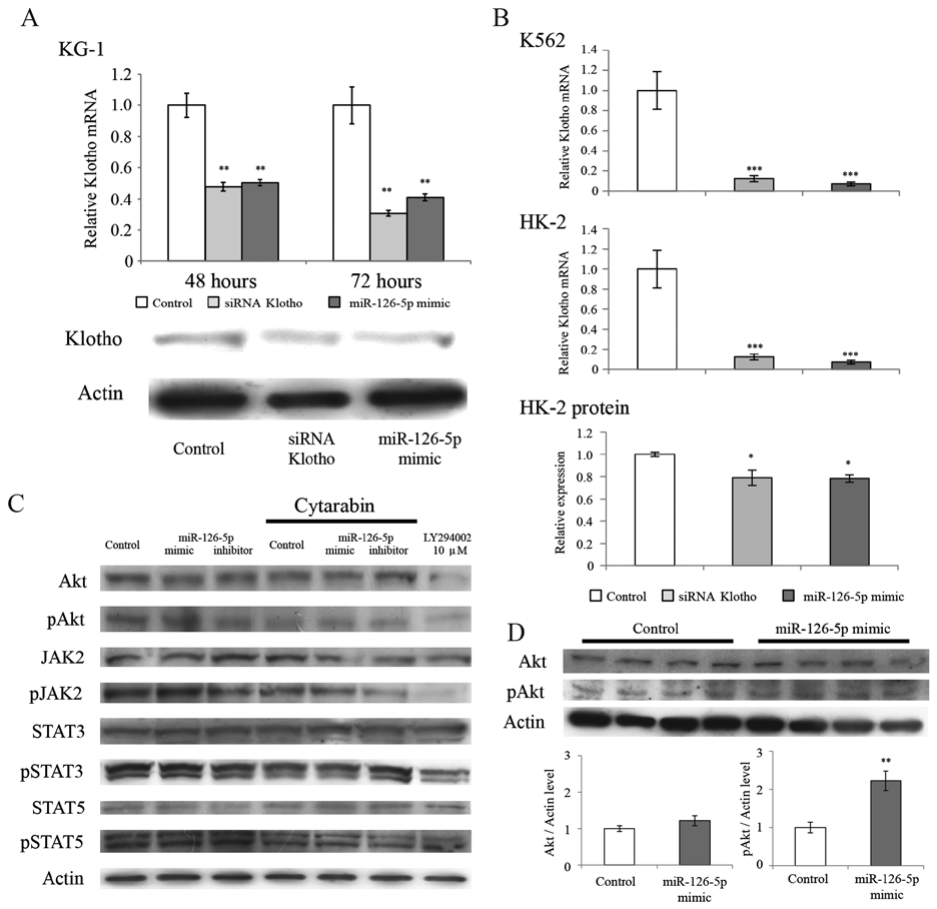
Effects of transfection with the miR-126-5p mimic on the expression of Klotho, Akt, and JAK/STAT kinases. (A) The relative level of the means and the standard error of *Klotho/Actin* expression is indicated. Cells were incubated for 48 or 72 h. *Klotho* mRNA expression levels were significantly decreased following transfection of the miR-126-5p mimic. A positive control, the siRNA of *klotho*, also significantly decreased *Klotho* mRNA levels. Comparisons between the 3 groups were performed with the Tukey-Kramer test, ^**^p<0.01 significantly different from the control group. Transfection of the miR-126-5p mimic also significantly decreased expression levels of the Klotho protein (relative expression level: control, 1.00±0.03; miR-126-5p mimic, 0.61±0.13; mean ± standard error, p=0.046, Student's t-test, n=3). This transfection did not affect the expression level of actin. (B) The miR-126-5p mimic or siRNA of *Klotho* was transfected into the K562 or HK-2 cells. The relative level of the means and the standard error of *Klotho/Actin* expression is indicated. The cells were incubated for 72 h. *Klotho* mRNA expression levels were also significantly decreased following transfection of the miR-126-5p mimic. Comparisons between the 3 groups were performed with the Tukey-Kramer test, ^*^p<0.05, ^***^p<0.001, significantly different from the control group. (C) Western blot analysis of Akt, the phosphorylated forms of Akt at serine 473 (pAkt), JAK2, the phosphorylated forms of JAK2 at tyrosine 1007 (pJAK2), STAT3, the phosphorylated forms of STAT3 at tyrosine 705 (pSTAT3), STAT5, the phosphorylated forms of STAT5 at tyrosine 694 (pSTAT5) and actin. The miR-126-5p mimic or inhibitor was transfected into KG-1 cells. The cells were incubated for 24 h and then administered cytarabin at a dose of 60 nM for 120 h. The PI3K inhibitor, LY294002 was administered at a dose of 10 *μ*M for 48 h as a positive control for the decline in the phosphorylation of Akt. (D) Western blot analysis of Akt, the phosphorylated forms of Akt at serine 473 (pAkt), and actin. The miR-126-5p mimic was then transfected into KG-1 cells. Transfection of the miR-126-5p mimic significantly increased the expression of the phosphorylated forms of Akt (relative expression level: control, 1.00±0.14; miR-126-5p mimic, 2.22±0.25; mean ± standard error, ^**^p<0.01, Student's t-test, n=4). This transfection did not affect the expression levels of actin and Akt.

The miR-126-5p mimic was transfected into the human cell lines, KG-1, K562 and HK-2. Transfection of the miR-126-5p mimic into the KG-1 cells resulted in decreased sensitivity to cytarabin, but not to idarubicin. Transfection of the miR-126-5p inhibitor did not alter the drug sensitivity (Table IV). The expression level of *Klotho* mRNA was decreased following transfection with the miR-126-5p mimic. Transfection of the miR-126-5p mimic also significantly decreased the expression levels of the Klotho protein (relative expression level; control, 1.00±0.03; miR-126-5p mimic, 0.61±0.13, mean ± standard error; p=0.046, Student's t-test, n=3). A typical western blot analysis is shown in Fig. 2A. The transfection of the miR-126-5p mimic as well as the positive control siRNA downregulated the expression of *Klotho* (Fig. 2A). The same actions were observed in the K562 and HK-2 cells (Fig. 2B). Azuma *et al* reported that *Klotho* mRNA and protein are abundantly expressed in HK-2 cells (30); thus, the transfection effect of miR-126-5p into HK-2 cells was also evaluated. Transfections of the miR-126-5p mimic into KG-1 or K562 cells as well as into the HK-2 cells downregulated the expression of *Klotho*. The expression level of *Klotho* mRNA was also decreased by the transfection with the miR-126-5p mimic in the K562 or HK-2 cells. Expression levels of the Klotho protein were decreased by the transfection with the miR-126-5p mimic in the HK-2 cells (Fig. 2B). Transfection of the miR-126-5p mimic resulted in an elevation in the phosphorylation of Akt in the KG-1 cells (Fig. 2C). Transfection of the miR-126-5p mimic also significantly increased the expression of the phosphorylated forms of Akt in the HK-2 cells (relative expression level: control, 1.00±0.14; miR-126-5p mimic, 2.22±0.25, mean ± standard error; p=0.005, Student's t-test, n=4; Fig. 2D). Transfection of the miR-126-5p mimic or inhibitor did not affect the expression or phosphorylation of JAK2, STAT3, STAT5 or Actin (Fig. 2C), or the mRNA expression of *ABCB1*, *BAALC*, *ERG*, *MN1*, *JAK2*, *WT1* or *ACTB* (data not shown).

## Discussion

The present study revealed two important issues: Firstly, higher levels of miR-126-5p/3p resulted in a poorer prognosis and secondly, miR-126-5p suppressed the expression of *Klotho* mRNA. One hundred and nine eligible patients who received intensive therapy were enrolled in the present study. Patients were divided into high and low groups based on their miR-126-5p expression levels. No significant differences were observed in the patient characteristics or the gene mutation statuses between the high and low groups. No significant differences were observed in the expression levels of gene-related abnormalities in AML between the two groups (Table III). These results suggest that the expression levels of the gene-related abnormalities were not related to prognosis in these groups.

The high expression group had a poorer prognosis than that of the low group (Fig. 1). The functions of miR-126-3p have been reported previously in an *in vitro* study; the miR-126-3p mimic was shown to inhibit cell apoptosis, increase cell viability and enhance proliferation and differentiation (7,16–19). These findings suggest that the upregulation of miR-126-3p is a predictor for poor prognosis. This was the first study to suggest that higher expression of miR-126-3p results in a poorer prognosis in AML patients. The present study showed that the high group had a poorer prognosis and an interaction was not observed between the hazard ratios of miR-126-5p and miR-126-3p (p=0.73). These results suggest that the upregulation of miR-126-5p may enhance the malignant potential of AML.

Recent studies have shown that miR-126-5p rescued the proliferation of endothelial cells by suppressing Dlk1, and decreased the proliferation of stromal cells (20–22). The overexpression of miR-126-3p/5p was shown to significantly induce apoptosis, and activate caspase-3 and caspase-7 by directly regulating ADAM9 and MMP7 in melanoma (23). These findings suggested that the upregulation of miR-126-5p resulted in opposing effects in different cell types; miR-126-5p inhibited proliferation in stromal cells, but activated proliferation in endothelial cells and inhibited apoptosis in melanoma cells. In the present study, the transfection of miR-126-5p into KG-1 cells induced drug resistance to cytarabin (Table IV) and the higher expression of miR-126-5p was associated with a poorer prognosis. The results of the present study revealed that the higher expression of miR-126-5p inhibited apoptosis induced by the cytarabin treatment, leading to a poor prognosis.

Bioinformatics, microRNA.org (http://www.microrna.org), is a comprehensive resource of microRNA target predictions and expression profiles. Target predictions are based on the development of a miRanda algorithm, which incorporates current biological knowledge on target rules and on the use of an up-to-date compendium of mammalian microRNAs (24). Klotho has been identified as an anti-aging gene, and also plays an important role in cancer tumorigenesis, cell survival, differentiation and metastasis (8). Wang *et al* reported that the knockdown of Klotho inhibited apoptosis, which significantly increased the expression of phospho-Akt and increased resistance to cisplatin in A549/DDP cells (11). A previous study reported that Klotho exerted an inhibitory effect on the IGF-1 pathway in both breast and pancreatic cancer cells (25–27). The PI3K/Akt pathway is one of the important downstream pathways of the IGF-1 pathway, and numerous studies have confirmed its role in the apoptosis of cancer cells (28–30). The present study demonstrated that miR-126-5p inhibited the expression of Klotho and upregulated the phosphorylation of Akt (Fig. 2). The present study and the previous findings collectively suggest that miR-126-5p may induce drug resistance by activating phospho-Akt, which targets Klotho.

In conclusion, the results of the present study demonstrated that the higher expression of miR-126-5p/3p in AML may result in a poor prognosis. Furthermore, miR-126-5p induced drug resistance to cytarabine by enhancing phosphorylation of Akt.

## Figures and Tables

**Table I:** Patient characteristics.

	miR-126-5p		P-value
	Low	High	
Gender (M/F)	29/25	31/24	NS
Age, years			
Mean	58.2	57.3	
Range	20–78	17–79	
Incipient (initial/relapse)	50/4	51/4	NS
FAB classification, n (%)			NS
M0	4 (7)	2 (4)	
M1	14 (26)	8 (15)	
M2	12 (22)	21 (38)	
M3	10 (19)	5 (9)	
M4	7 (13)	10 (18)	
MDS	5 (9)	1 (2)	
Other	2 (4)	8 (14)	

A statistical analysis for single comparisons was performed using a two-tailed χ^2^ test. Comparisons between 2 groups were analyzed with the Mann-Whitney U test. NS, not significant; M, male; F, female.

**Table II:** Gene mutation status.

Gene	miR-126-5p		P-value
	Low, n (%)	High, n (%)	
*NPM1*	13 (24)	12 (22)	NS
*PML-RARA*	10 (19)	5 (9)	NS
*CEBPA*	13 (24)	7 (13)	NS
*CBFb-MYH11*	2 (4)	6 (11)	NS
*FLT3-ITD*	6 (11)	1 (2)	NS
*FLT3 D835*	1 (2)	2 (4)	NS
*N-RAS* (codon 12/13)	3 (6)	3 (5)	NS
*K-RAS* (codon 12/13)	1 (2)	1 (2)	NS
Other^a^	1 (2)	7 (13)	

A statistical analysis for single comparisons was performed using a two-tailed χ^2^ test or Fisher’s exact test (expected frequency <5). NS, not significant.

a The gene mutation of *BCR-ABL* was observed in the low expression group.

**Table III:** Relative expression levels of the gene-related abnormalities in AML.

Gene	miR-126-5p		P-value
	Low	High	
*ABCB1*	0.055±0.128	0.027±0.061	NS
*BAALC*	0.589±1.836	0.927±2.034	NS
*ERG*	0.251±0.346	0.184±0.190	NS
*MN1*	0.137±0.493	0.117±0.215	NS
*JAK2*	0.583±0.813	0.400±0.525	NS
*WT1*	2,989±4,832	2,257±2,511	NS

Values are represented as the mean ± standard deviation. Comparisons between the 2 groups were performed with the Mann-Whitney U test. NS, not significant. Relative expression levels were evaluated using glucose-phosphate isomerase (*GPI*) as a reference gene.

**Table IV:** Effects of the mimic or inhibitor miR-126-5p transfection on drug sensitivity in the KG-1 cells.

	NC	miR-126-5p	
		Mimic	Inhibitor
Idarubicin	38.0±1.6	43.8±2.4	46.0±1.6
Cytarabin	35.1±7.1	54.0±5.3^a^	36.6±2.5

The control group was transfected with negative control RNA. Values are represented as the mean ± standard error. Comparisons with the negative control group were performed with the Tukey-Kramer test.

a p<0.05. NC, negative control.
